# War Injuries of the Jaws

**Published:** 1918-08

**Authors:** 


**War Injuries of the Jaws**. Norman G. Bennett. *The Practitioner,* No. 591, Vol. XCIX, No. 3.

The first consideration is hemorrhage, but this applies chiefly to cases seen shortly after injury. The lingual or ramus artery, or branches of them, may bleed freely.

The points that next demand attention are support of fractured portions of mandible and abatement of sepsis.

It is best to make use a of light aluminium chin support, held in place by a bandage. Undue pressure may damage the soft parts and promote suppuration and formation of sinuses.

As soon as possible, all loose teeth, broken teeth, foreign bodies, loose pieces of dead bone, and septic roots should be removed, as well as the teeth in the immediate vicinity of the fracture.

There are, however, exceptions to this rule and judgment is required.

As long as free suppuration around the ends of the bone continues, usually with sinuses opening externally, no process of repair can even begin.

The external wound requires ordinary antiseptic dressing treatment.

The objects ultimately to be attained are :

Firm union of fractured portions of bone,

Restoration of the jaws as nearly as possible to normal.

Replacement of lost portions by prothetic appliance.

Union of the soft tissues, with as little contraction and scarring as possible, by immediately suturing or gradual healing by granulation, or with the aid of subsequent plastic operations.

It will be found that most fractures of the mandible sort themselves into three main groups :

The first group consists of those cases in which the anterior portion of the mandible with the teeth contained therein as far back as the premolars, or even the molars, is destroyed.

The second group includes cases of fracture in the premolar or molar region anterior to the masseter.

The third group consists of fractures at the angle, or of the ascending ramus, or coronoid process, or even of the neck of the condyle.

Fracture at the angle is common, and is often associated, when caused by a bullet passing in an oblique direction, with fracture in the premolar region of the opposite side.

In some cases of double or triple fracture, the jaw is so severely comminuted that the various parts are quite loose.

Injuries of the maxilla can scarcely be classified. They may affect any part of it.

The amount of bone destroyed determines, to some extent, the ultimate result to be attempted, and, therefore, the methods to be employed.

It will generally be agreed that with loss of bone up to half an inch in length, bony union may be expected with some confidence. Beyond three-quarters of an inch replacement by natural growth cannot be expected, and the initial course of treatment is to some extent determined by the intention to make use of a bone graft ultimately.

With small destruction of bone in cases seen soon after injury, immediate methods may be adopted; but with a larger amount of destruction, or in old standing cases, or after division for false union. gradual methods are preferable.

It is now generally agreed that the growth of new bone is not dependent only on the periosteum. Bone may grow outwards from the medullary substance of the fractured ends, or may even be reproduced from small comminuted pieces, lodged between the fractured ends. On this account it is important that sepsis should be controlled as soon as possible after injury, and that no bone should be removed that does not separate naturally as a necrotic fragment.

The cases in which bony union cannot be hoped for without the aid of a bone-graft, demand much consideration. Even in these cases, the writer thinks that the fragments should be reduced to normal positions. If a bone-graft is ultimately successful, well and good; if not, a fibrous union must be accepted, and will probably result in a more satisfactory mandible than if the parts had been allowed to contract with the object of getting bony union.

It is unfortunately true that contraction of the mandible or deviation to one side, so alters the relationship of the alveolar arches as to cause much difficulty in the fitting of artificial dentures, and to impair their efficiency.

There is, however, one form of displacement that materially assists bony union and does not cause loss of occlusion at any moment. In those cases of unilateral fracture through the molar region the larger fragment deviates toward the injured side. This displacement can be corrected, leaving a space between the fractured ends.

Consideration of the usual types of displacement, associated with the different positions of fractures makes it obvious that the treatment will vary greatly. When the fracture is behind the anterior border of the masseter, it is usually sufficient to keep the mandible closed in normal occlusion by suitable bandaging. In fractures of the ascending ramus the elevating muscles on the injured side cause an upward tilt on that side with separation of the teeth on the uninjured side. This can be corrected by gagging the teeth on the injured side and applying upward pressure externally to the mandible. In cases of fracture high in the ascending ramus near the neck of the condyle, complete immobilization is required until the fracture is partly consolidated.

Almost all fractures anterior to the masseter involve displacement. Gradual reduction, and immobilization following immediate or gradual reduction, usually involve the use of some more or less complicated and specially-made appliance.

As for the appliances most useful for reduction and immobilization, the simplest form consists of a cast metal cap splint, fixed with cement to the teeth, and holding the parts in their normal positions.

In simple cases, the well known wire Hamond splint may be used.

Northcroft has devised an ingenious and simple splint of metal. It covers the lingual surface of the teeth, and at the necks of the teeth is provided with upturned lugs; wires passed between the teeth engage these lugs, and the ends are twisted together on the buccal surface.

For cases of multiple fracture of the mandible a simple vulcanite lingual splint perforated with holes, for the passage of bronze wires, by means of which the teeth are ligatured in position to occlude with the maxillary teeth, is very efficient.

The second group of appliances includes those used generally when lateral deviation of half the jaw has occurred. For this purpose, Payne's cradle splint is most commonly employed.

The appliances of the third group are used mainly for correction of displacement of the maxilla. Such appliances are applied firmly to the head by means of an encircling band passing round the forehead; strong wire connections brought round the sides of the face afford a means of giving stability to an intra-oral splint.

Among the most difficult cases are those of fracture with loss of bone in the molar region on one side in which the smaller fragment contained no teeth. It is usually best to begin treatment by immobilizing the larger fragment.

When lateral deviation of the jaw has been corrected, and union of fractured parts has progressed to some extent, it is often possible to use an appliance that will allow the patient some use of his jaw ar.d yet prevent deformity.

Bone-grafting should not be performed until all suppuration and separation of sequestra have ceased and the parts are completely healed; the two portions of the mandible must be fixed absolutely firmly in their correct relative positions by means of an intra-oral splint; in the operation itself, all communication with the oral cavity must be avoided.

The value of massage consists in the prevention of contraction and the softening of cicatrices, and in the diminution of stiffness a.ound the temporo-mandibular articulation.

